# Postoperative outcomes after off-label cryoanalgesia during minimally invasive repair of pectus excavatum in children younger than 12 years

**DOI:** 10.3389/fped.2026.1857143

**Published:** 2026-06-17

**Authors:** Alberto Jarrin Lopez, Hanmin Lee, Sunghoon Kim

**Affiliations:** 1 Department of Surgery, University of California, San Francisco – East Bay, Oakland, CA, United States; 2 Surgical Innovations Program, Department of Surgery, University of California San Francisco, San Francisco, CA, United States; 3 University of California, San Francisco–Stanford Pediatric Device Consortium, San Francisco, CA, United States; 4 Division of Pediatric Surgery, University of California, San Francisco Benioff Children's Hospitals, San Francisco/Oakland, CA, United States

**Keywords:** cryoablation, minimally invasive repair of pectus excavatum, pectus excavatum, postoperative pain, real world evidence (RWE)

## Abstract

**Background:**

Intraoperative cryoanalgesia has been widely adopted to reduce postoperative pain, opioid use, and length of stay after minimally invasive repair of pectus excavatum (MIRPE). However, its use in children younger than 12 years remains off-label, and outcomes in this age group are poorly characterized. This study evaluated postoperative outcomes after cryoanalgesia during MIRPE in carefully selected children younger than 12 years.

**Methods:**

We performed a single-center retrospective chart review of patients younger than 12 years who underwent MIRPE with thoracoscopic transthoracic cryoanalgesia between August 2019 and July 2023. Patients receiving epidural analgesia or patient-controlled analgesia were excluded. Primary outcomes were inpatient opioid consumption, converted to oral morphine equivalents per kilogram (OME/kg), and hospital length of stay. Secondary outcomes included inpatient pain scores, postoperative sensory symptoms, and complications.

**Results:**

Ten patients met inclusion criteria. The mean age was 10.1 ± 0.9 years, and 70% were male. Median total inpatient opioid consumption was 0.96 OME/kg (IQR, 0.47–1.17), and median length of stay was 2 days (IQR, 2–3). Median inpatient pain scores decreased from 2 (IQR, 0–4) on postoperative day 0 to 0 (IQR, 0–2) on postoperative day 1. Two postoperative complications occurred; neither was directly attributable to cryoanalgesia. Among patients with available follow-up, no persistent pain, paresthesia, or numbness was reported by 9 months postoperatively.

**Conclusions:**

Off-label cryoanalgesia during MIRPE in children aged 9–11 years was feasible and associated with low opioid use, acceptable pain control, short length of stay, and no cryoanalgesia-related complications. Larger prospective studies are needed.

## Introduction

The minimally invasive thoracoscopic repair of pectus excavatum (MIRPE), commonly referred to as the Nuss procedure, is the standard approach for correction of the concave chest wall deformity. The procedure is inherently painful due to forced chest wall remodeling with substernal bar placement, resulting in rib and costochondral stress as well as intercostal nerve irritation. Accordingly, a patient's ability to tolerate pain in the acute and subacute postoperative period is a limiting factor in performing the operation.

Since University of California, San Francisco (UCSF) surgeons first described the use of thoracoscopic, transthoracic cryoanalgesia during the Nuss procedure in 2016, this technique has been validated both internally and externally, including in randomized controlled trials demonstrating decreased length of stay (LOS) and reduced opioid requirements (1–8). In December 2020, AtriCure received FDA label expansion to formally include pediatric patients older than 12 years within the indicated use of cryoablation (9).

Despite widespread adoption of this technique, the existing literature is limited to patients older than 12 years at the time of operation, with a reported median age of 14 years (range, 1–31) (10). However, the Nuss procedure is also performed in younger patients under select circumstances, such as severe restrictive lung disease or cardiac compression that cannot be delayed, rapidly progressive deformity in which early intervention may prevent severe asymmetry, and cases of significant psychological distress prior to the typical adolescent window for repair.

The use of cryoanalgesia in patients younger than 12 years is therefore off-label, reflecting the absence of dedicated pediatric pivotal trials, limited multicenter data in this age group, and incomplete characterization of long-term sensory outcomes after intercostal nerve cryoablation in skeletally and neurologically developing children. Accordingly, in our institution, off-label cryoanalgesia has been used in children younger than 12 years undergoing the Nuss procedure for these indications. In this study, we evaluate postoperative outcomes associated with intraoperative cryoanalgesia in children younger than 12 years, with particular focus on opioid consumption, length of stay, pain scores, and complications.

## Methods

With approval from the institutional review board at UCSF Benioff Children's Hospital (IRB #25–44167), we conducted a retrospective chart review of patients who underwent the Nuss procedure with cryoanalgesia (cryoICE; AtriCure, Inc., West Chester, OH, USA) between August 2019 and July 2023. The requirement for study-specific informed consent was waived because of the retrospective design and use of deidentified data. Inclusion criteria included age <12 years at the time of surgery and absence of epidural analgesia or patient-controlled analgesia (PCA). All procedures were performed by a single attending pediatric surgeon. The technical details of the Nuss procedure and the application of cryoanalgesia have been previously described and were consistently applied to all patients (11, 12).

Operative candidacy for the Nuss procedure under 12 years of age was determined on an individualized basis by the operating surgeon in consultation with the multidisciplinary team, and required at least one of the following: severe cardiopulmonary compromise that could not be safely delayed, rapidly progressive asymmetric deformity, or substantial psychosocial impairment for which earlier repair was felt to be in the patient's best interest. Candidacy for adjunctive cryoanalgesia required, in addition, thoracic dimensions sufficient to accommodate safe transthoracic cryoprobe placement under thoracoscopic visualization without compromising the operative corridor, and anticipated tolerance of brief single-lung ventilation. Patients in whom either of these considerations was judged to be prohibitive were not offered cryoanalgesia and were therefore excluded from this cohort. No age-specific modifications were made to the cryoanalgesia technique itself; standard probe temperatures, freeze durations, and treated intercostal levels were applied as previously described in our prior reports (1, 11, 12).

Postoperatively, patients were initially monitored in the post-anesthesia care unit and subsequently transferred to a standard inpatient unit. Patients received intravenous opioids (hydromorphone or morphine) or oral opioids (oxycodone) as needed for breakthrough pain. All administered medications were recorded in the medication administration record per institutional protocol. Inpatient pain levels were documented numerically by nursing staff. Following discharge, patients were evaluated in clinic at approximately 1–2 weeks, 2–3 months, and 1 year postoperatively, with additional visits as clinically indicated.

Patient characteristics were summarized descriptively. Age and weight were reported as means with standard deviations, while all other continuous variables were reported as medians with interquartile ranges given the small sample size and non-normal distribution. The primary outcome were postoperative opioid use and hospital length of stay (LOS), measured in days. The former included total opioid consumption during hospitalization and daily opioid use by postoperative day. All opioid doses (intravenous and oral) were converted to oral morphine equivalents (OME) using standard equianalgesic conversion factors (e.g., intravenous morphine × 3, intravenous hydromorphone × 20, oral oxycodone × 1.5), consistent with published CDC guidance and institutional pharmacy references. Opioid consumption was normalized to body weight and expressed as OME per kilogram (mg/kg) to account for differences in patient size.

Secondary outcomes included postoperative pain (inpatient and outpatient); and procedure-related complications. Pain scores were assessed using a 10-point numeric rating scale and abstracted from the medical record. All data were recorded and managed using Microsoft Excel for Mac, version 16.103.4 (Microsoft Corporation, Redmond, WA).

## Results

Table 1 summarizes the demographic characteristics of the study cohort. The majority of patients (70%) were male, with a mean age of 10.1 ± 0.9 years (range, 9–11).

**Table 1:** Demographics.

Variable	Value
Number of patients, n	10
Sex (n) Male Female	7 3
Age, years	10.1 ± 0.9 (range 9–11)
Height, cm	147.4 ± 10.1 (range 135–167)
Weight, kg	36.4 ± 10.8
BMI, kg/m^2^	15.6 (IQR 14.1–17.6)
Haller Index	3.8 (IQR 3.32–4.1)

### Primary outcome: opioid use and length of stay

**Figure 1:**
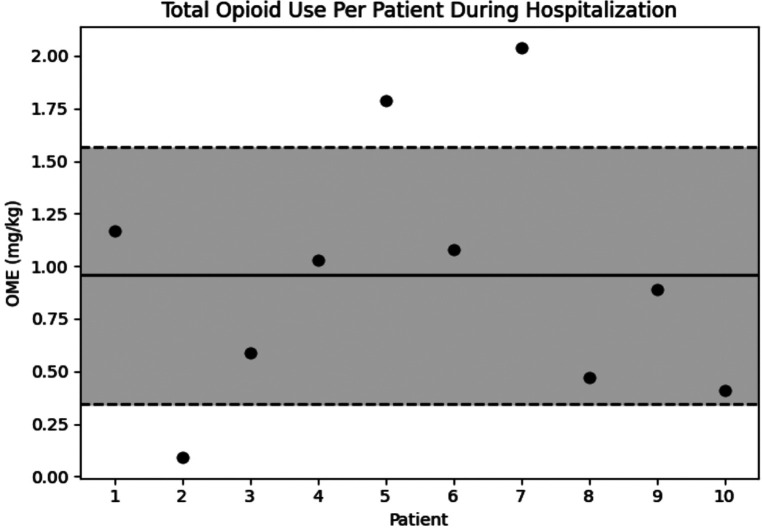
Total opioid use during hospitalization is presented as oral morphine equivalents (OME, mg/kg) per patient. Each point represents an individual patient. The solid line indicates the mean OME, and the dashed lines represent ± 1 standard deviation (SD). The shaded region corresponds to the SD range.

**Figure 2:**
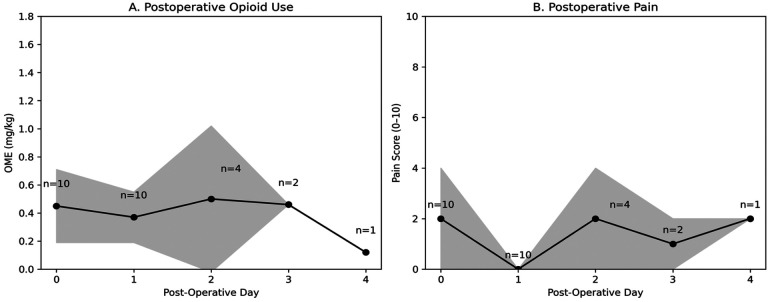
**(A)** Postoperative opioid use is presented as mean oral morphine equivalents (OME, mg/kg) per patient per day with standard deviation (SD). **(B)** Postoperative pain is presented as median pain score (0–10 scale) with interquartile range (IQR). For both panels, the number of patients contributing data at each postoperative day is indicated. The decreasing sample size over time reflects patient discharge, with later time points representing a subset of patients with prolonged hospitalization.

Opioid use was summarized as both total consumption and daily use by postoperative day (Table 2). Three outliers were identified. Patient 2, who was discharged on postoperative day (POD) 1, did not require any postoperative opioids. In contrast, Patients 5 and 7, who were discharged on POD 4 and POD 3, respectively, had the highest opioid consumption during hospitalization (Figures 1, 2A). The median length of stay (LOS) was 2 (IQR 2–3) days.

**Table 2:** Postoperative opioid use (OME) and length of stay (LOS).

Outcome	Overall	POD 0	POD 1	POD 2	POD 3	POD 4
Patients (n)	10	10	10	4	2	1
Total OME, mg/kg [median (IQR)]	0.96 (0.47–1.17)	—	—	—	—	—
Daily OME, mg/kg [median (IQR)]	—	0.45 (0.28–0.71)	0.37 (0.21–0.48)	0.23 (0.18–1.08)	0.46	0.12
LOS, days [median (IQR)]	2 (2–3)	—	—	—	—	—

Values are presented as median (IQR). For postoperative days with *n* = 1, only the observed value is shown. Decreasing sample size reflects hospital discharge.

### Secondary outcomes: pain and complications

Median inpatient pain scores decreased from 2 (IQR 0–4) on POD 0 to 0 (IQR 0–2) on POD 1. Among patients who remained hospitalized, median pain scores were 2 (IQR 0–5) on POD 2. Estimates for later postoperative days were limited by small sample sizes. Figure 2B illustrates pain scores by postoperative day.

Supplementary Table S1 summarizes long-term postoperative symptoms, including pain, paresthesia, and numbness. Given variability in follow-up timing, visits were grouped into four intervals: 0–4 weeks, 4–12 weeks, 3–9 months, and ≥9 months. By 9 months postoperatively, no patients reported persistent pain, paresthesia, or numbness among those with available follow-up.

Supplementary Table S2 summarizes postoperative complications. Two complications were identified (2/10, 20%), one of which was pain-related. A 10-year-old patient with a history of Ehlers–Danlos syndrome, who had minimal pain during hospitalization and at discharge, presented to the emergency department on the day of discharge (POD 1) with chest tightness. The patient was worked up to rule out cardiopulmonary etiologies, readmitted overnight for observation, and discharged the following day with adequate pain control (POD 2).

## Discussion

In this single-center, retrospective study of patients undergoing MIRPE, we report outcomes associated with the use of off-label cryoanalgesia. To our knowledge, this is the first report describing opioid utilization after MIRPE in patients aged 9–11 years. Although our study is limited by the absence of a direct comparison group, opioid consumption can still be contextualized by relating it to (a) other studies and (b) related outcomes such as postoperative pain, symptoms, and length of stay. The use of cryoanalgesia in this age group remains off-label because dedicated pediatric pivotal data are not yet available, multicenter experience in patients under 12 years is limited, and longer-term sensory follow-up after intercostal nerve cryoablation in skeletally and neurologically developing children has not been fully characterized. The 2020 FDA label expansion was supported by data drawn from patients aged 12 years and older (9). The rationale for off-label use in younger children therefore rests on three considerations: (1) extrapolation from the now-substantial adolescent and adult experience, including randomized data demonstrating reduced opioid use and length of stay (2–8). (2) the technical similarity of the cryoablation step across age groups when thoracic anatomy permits; and (3) the clinical imperative to provide effective, opioid-sparing analgesia in younger patients in whom the Nuss procedure is undertaken for severe cardiopulmonary, anatomic, or psychosocial indications. Reporting on outcomes in this group is intended to inform that risk–benefit balance until prospective pediatric data become available.

The median postoperative OME/kg consumed until discharge was 0.96 (0.47–1.17) OME/kg, with a mean of 0.96 ± 0.61 OME/kg. The median was favored for reporting given the small sample size and skewed distribution, although the median and mean were similar. Within the published literature, two studies describe inpatient OME/kg as a median, with reported values of 2.3 (1.2–3.1) OME/kg and 1.1 (0.5–2.2) OME/kg (5, 13). Lai et al. reported total opioid consumption as a mean of 2.8 ± 2.5 OME/kg (3). In these three studies, adjunct epidural analgesia or PCA was used. Our median (and mean) value of 0.96 OME/kg is therefore concordant, given that patients in our cohort did not receive adjunct epidural analgesia or PCA.

Direct comparisons between cryoanalgesia and epidural analgesia in the pediatric Nuss population have been previously reported. Graves and colleagues demonstrated that intraoperative cryoanalgesia reduced opioid requirements and shortened length of stay compared with epidural analgesia in a randomized clinical trial (2), and a prior single-institution series from our group similarly reported reduced opioid use after cryoanalgesia relative to historical epidural practice (12). A subsequent cohort study by van Braak et al. found cryoablation to be at least equivalent to epidural analgesia, with a more favorable side-effect and mobility profile (6). At our own institution, epidural analgesia was the routine analgesic adjunct for the Nuss procedure prior to the introduction of cryoanalgesia in 2016; since that time, epidural use has been largely abandoned for this operation, and the patients in the current cohort received neither epidural analgesia nor PCA. Although a contemporaneous within-institution comparison is not available in this series, this practice transition itself reflects our institutional experience that cryoanalgesia provides at least equivalent analgesia with a more favorable recovery profile.

The median inpatient pain score was 2 on a 10-point scale, and median length of stay was 2 (2–3) days. These metrics are comparable to published values in patients older than 12 years who received cryoablation for the Nuss procedure (2–7). Regarding long-term symptoms, all patients were free of pain, paresthesia, and numbness by nine months postoperatively. The types and frequencies of symptoms reported in our cohort after cryoablation are consistent with those reported in the literature for children older than 12 years (14).

Among our cohort of 10 patients, 2 experienced complications. The first had a complication unrelated to cryoablation: the pleura was inadvertently punctured with a crossbar by a supervised trainee surgeon. The second complication was a same-day return to the hospital after discharge for symptoms of “chest tightness,” despite minimal pain at discharge on POD 1. The patient was admitted for observation, provided reassurance, and discharged on POD 2 with adequate pain control and adequate symptom expectations. The chest tightness was attributed to bar placement and was explained as an expected postoperative symptom. Overall, there were no complications directly attributable to the cryoablation itself, either from inadvertent injury caused by the cryoprobe to the surrounding anatomy or from a technical failure to ablate the appropriate intercostal nerves. In this regard, cryoablation did not appear to contribute to complications in our series.

## Limitations

Our study has several important limitations. First, the sample size of 10 patients is small, which precludes formal statistical inference, limits external generalizability, and prevents pre-specified subgroup analyses—most notably stratification by number of bars placed, which is known to influence postoperative pain after the Nuss procedure. Second, there is no contemporaneous control group; comparisons in this report are anchored to historical adolescent and adult cohorts from the published literature and to our institution's pre-2016 epidural-based practice, both of which differ from the current cohort in age, era, and adjunctive analgesia. Third, longitudinal pain data after the first one to two inpatient days are limited by sample attrition as patients were discharged, and structured long-term sensory follow-up beyond nine months was not consistently captured across all patients. Fourth, the entire cohort was operated upon by a single attending pediatric surgeon at a single tertiary center, which may limit external generalizability of both the technical findings and the analgesic outcomes. These limitations are partially mitigated by the use of standardized, weight-adjusted opioid metrics (OME/kg), conversion of all opioid types to a common unit, and a uniform surgical and analgesic protocol applied to all patients. Multicenter, prospective studies with matched comparison groups, pre-specified subgroup analyses (including by number of bars), and standardized long-term sensory follow-up are needed to confirm the safety and efficacy of cryoanalgesia in children younger than 12 years and to define the optimal indications and technique in this population.

## Conclusion

In summary, our findings suggest that off-label cryoanalgesia during MIRPE in children aged 9–11 years is feasible and was associated with low postoperative opioid use, acceptable pain control, and no complications directly attributable to the cryoablation technique. Although larger comparative studies are needed, these early results support the potential safety and utility of cryoanalgesia in carefully selected patients younger than 12 years.

## Data Availability

The raw data supporting the conclusions of this article will be made available by the authors, without undue reservation.
