# Supplementary figures and images for: Postoperative outcomes after off-label cryoanalgesia during minimally invasive repair of pectus excavatum in children younger than 12 years
Source: Front Pediatr. 2026 Jun 17;14:1857143. doi: 10.3389/fped.2026.1857143 (PMC13318872; doi:10.3389/fped.2026.1857143)

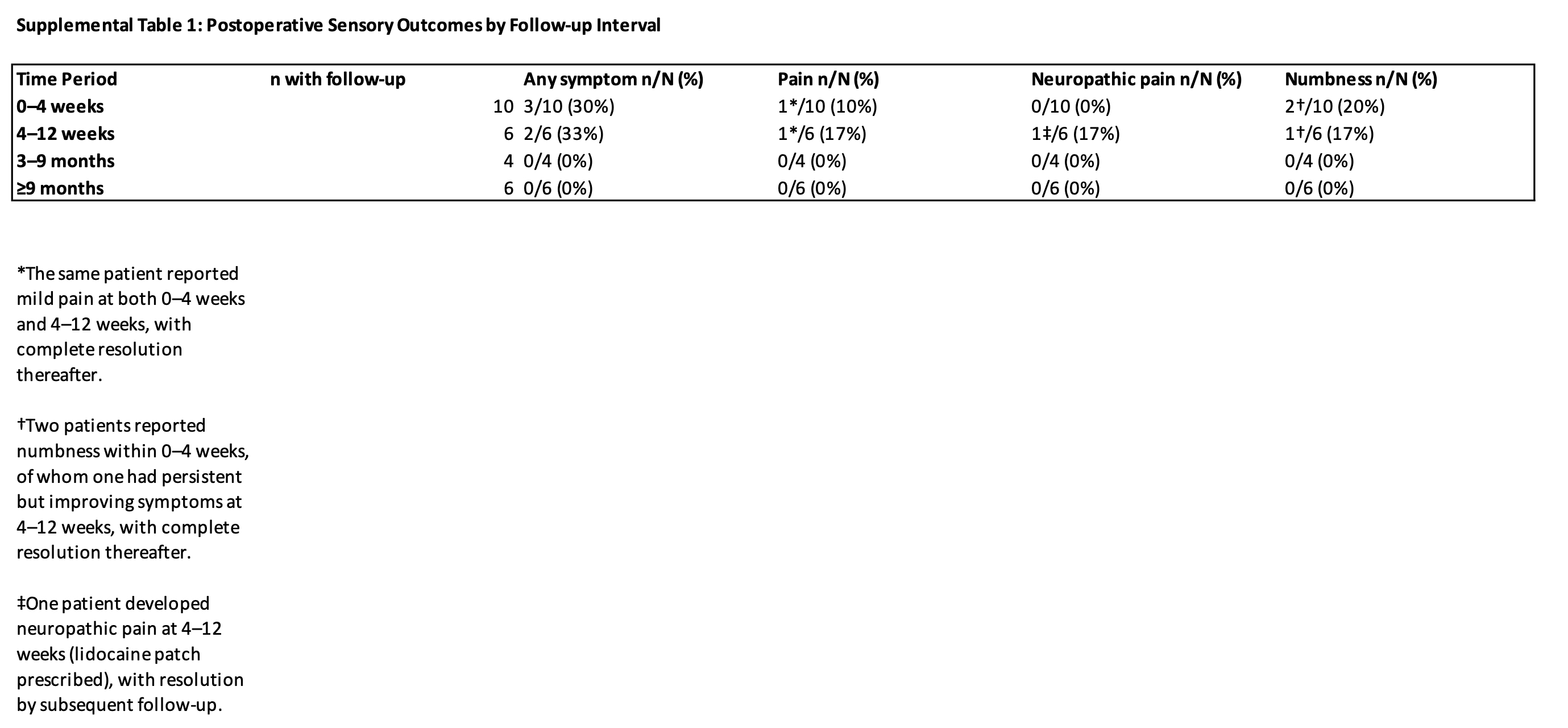

Supplement: Supplementary file 1 [file Image1.jpeg]

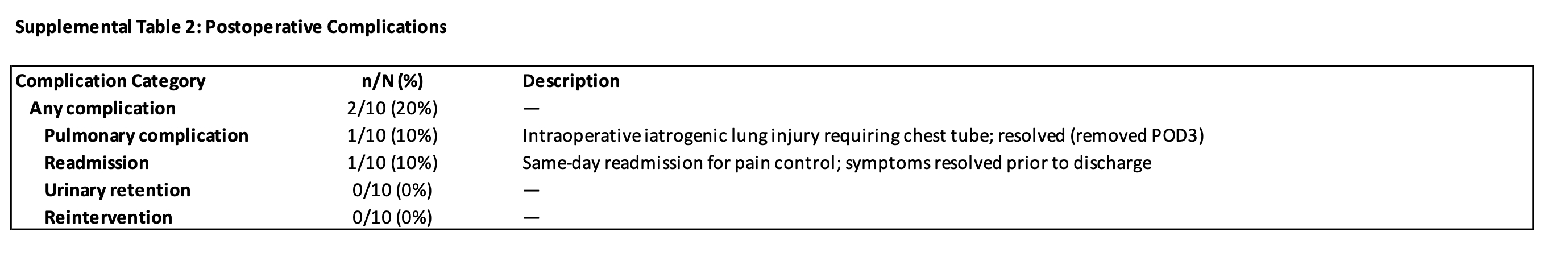

Supplement: Supplementary file 2 [file Image2.jpeg]
